# Development of a Core Set of Quality Criteria for Virtual Reality Applications Designed for Older Adults: Multistep Qualitative Study

**DOI:** 10.2196/45433

**Published:** 2023-09-27

**Authors:** Alina Napetschnig, Klara Brixius, Wolfgang Deiters

**Affiliations:** 1 Department of Community Health Hochschule für Gesundheit Bochum Bochum Germany; 2 Institut für Kreislaufforschung und Sportmedizin Deutsche Sporthochschule Köln Cologne Germany

**Keywords:** virtual reality, older adults, quality criteria, user-centered

## Abstract

**Background:**

Virtual reality (VR) applications are gaining growing significance, particularly among older adults. These applications can provide valuable support to older adults by offering immersive VR content that positively influences various aspects of their daily lives, including activities of daily living. Furthermore, VR applications can contribute to the enhancement of cognitive and motor skills, ultimately leading to an improved quality of life for older individuals. Nevertheless, to ensure a positive impact, it is crucial to develop VR experiences that are tailored to the needs and preferences of the users.

**Objective:**

This study aims to develop a core set of quality criteria and guidelines for the development of user-centered VR applications specifically designed for older adults (target group).

**Methods:**

The multistep qualitative study design comprised several key stages, beginning with a systematic literature search. This was followed by a framework analysis aimed at identifying a core set of criteria. Subsequently, these criteria underwent validation through expert workshops. The outcomes achieved through this iterative process were organized and categorized into criteria, accompanied by explanations detailing the underlying categories or codes.

**Results:**

The quality criteria core set for older adults–friendly VR applications has been developed through an iterative process. It is divided into 2 distinct parts, each containing criteria categorized into specific areas. The first part includes the following categories: (1) quality assurance of medical/health content, (2) data protection provisions, (3) quality requirements, (4) consumer protection, and (5) interoperability. The second part includes the following categories: (1) graphic/quality, (2) 3D character/avatar, (3) providing in-game instructions and prompts, (4) interaction, (5) navigation, and (6) promotion of user motivation and loyalty to use. The results imply a differentiated scope as well as a differentiated granularity of the criteria.

**Conclusions:**

Considering the ongoing advancement of VR technology and the diverse needs within the older adult demographic, it is essential to assess the quality criteria core set results on an individual basis.

## Introduction

**Figure 1:**
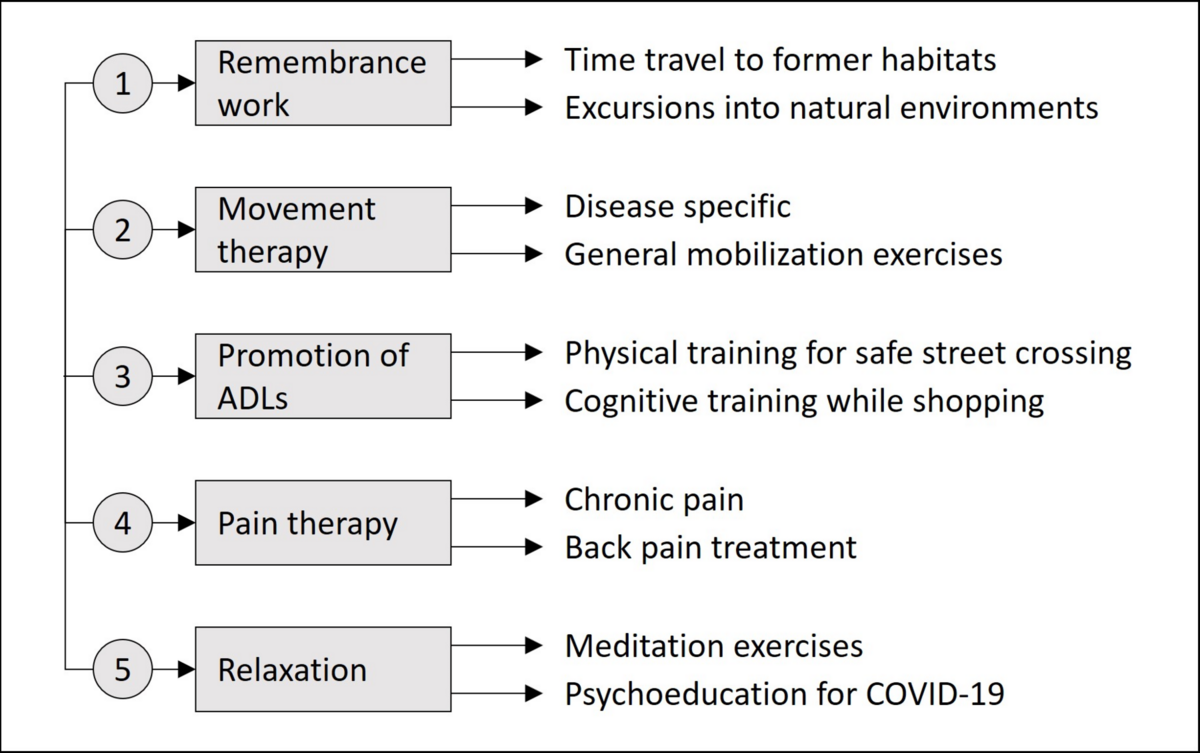
Fields of application and exemplary scenarios in the context of virtual reality and seniors. ADLs: activities of daily living.

Virtual reality (VR) goggles are a technology that has been gaining increasing popularity in recent years, and their functionality continues to expand [1]. VR goggles are a technical medium that allows immersion in VR [2]. VR stands for computer-generated environments that enable people to experience and interact in digital worlds [3]. VR applications have found their place in various fields. While they are already a popular choice for leisure, especially among the younger generation, they are increasingly employed in health care, often in the form of “serious games,” to address specific human conditions [1,4]. VR applications offer numerous benefits, including use among older adults [2,5,6]. VR applications are intended to specifically promote the competencies of the older adults and to help them achieve more independence in old age [7]. Figure 1 illustrates various application fields in VR for older adults, along with 2 existing exemplary scenarios.

Although many interventions have been shown to improve the health status of older adults through the use of VR, there remains a need for research to determine usability and acceptance factors [8]. Furthermore, attention should be paid to target group-specific development [9]. Currently, VR applications in the health care context are typically developed through collaboration between VR developers or computer scientists on the technical side and medical staff or health scientists on the content side [10]. The challenge stems from the unfamiliarity between VR developers and medical personnel, hindering the establishment of comprehensive VR development requirements. Additionally, user-centered development is increasingly complex due to the growing heterogeneity in age-related factors among older adults [6,7,11,12].

VR development for older adults should promote healthy aging and enhance quality of life. The World Health Organization (WHO) describes healthy aging as a process of developing and maintaining the functional capacity that enables well-being in old age [13]. Under the aspect of “healthy aging,” the WHO has defined various influencing factors that can have a positive effect on the aging process. These include factors that go beyond the elimination of diseases. As a result, active health promotion throughout one’s life and targeted support measures to maintain functional capacity in old age are essential. The term health, in the context of healthy aging, is a person’s ability to perform or pursue those things that they prioritize [14]. To fulfill these goals as much as possible, the needs of older adults must be taken into account. At the same time, the feasibility of continuous development of VR must be addressed [15]. Guidelines and best practice frameworks can assist in using VR applications effectively for specific goals [16]. Guidelines developed through collaborative research with experts in VR development and older adult–focused VR applications can serve as an initial orientation guide for tailored and beneficial VR applications for older adults.

This study aimed to establish a core set of quality criteria for older adult–friendly VR applications, which could serve as guidelines for similar target-oriented VR development. It involved considering criteria relevant to older adult–oriented VR application development.

## Methods

### Overview

Three different approaches were combined in this study. First, a systematic literature review search (SLS) was performed on April 7, 2021, to obtain an overview of existing guidelines or development recommendations for VR applications. The results mainly considered studies on VR application development and technology development guides providing recommendations. Second, the results were clustered in a framework analysis to systematize criteria or categories. Finally, in the third step, expert workshops were conducted with VR development experts and VR application experts with reference to the target group of older adults to check or adapt the validity of the elaborated results.

### Systematic Literature Search

To align with the study’s objectives, we formulated the following research question: *What universal criteria exist for development recommendations for VR applications and what are their contents?*

The following 3 main keyword groups were examined for the development of the search strategies:

1. (universal) criteria and corresponding synonyms (eg, features, characteristics, features, requirements, quality criteria);
2. development recommendations and corresponding synonyms (eg, development tips, hints, advice, suggestions, design, framework, evaluation); and
3. VR (applications) and corresponding synonyms (eg, virtual system, VR goggles, VR technology, VR head-mounted display, VR headset).

For the 3 groups of keywords, the thesaurus was also reviewed. Second, a search strategy for each database was developed. The detailed strategy for PubMed is shown in Textbox 1. Specific search strategies are outlined in Table 1.

**Search string used for the PubMed search.**

((“criteria s”[All Fields] OR “criterias”[All Fields] OR “standards”[MeSH Subheading] OR “standards”[All Fields] OR “criteria”[All Fields]) AND ((“develop”[All Fields] OR “develope”[All Fields] OR “developed”[All Fields] OR “developer”[All Fields] OR “developer s”[All Fields] OR “developers”[All Fields] OR “developing”[All Fields] OR “developments”[All Fields] OR “develops”[All Fields] OR “growth and development”[MeSH Subheading] OR (“growth”[All Fields] AND “development”[All Fields]) OR “growth and development”[All Fields] OR “development”[All Fields]) AND (“recommend”[All Fields] OR “recommendable”[All Fields] OR “recommendation”[All Fields] OR “recommendation s”[All Fields] OR “recommendations”[All Fields] OR “recommended”[All Fields] OR “recommending”[All Fields] OR “recommends”[All Fields])) AND (“virtual reality”[MeSH Terms] OR (“virtual”[All Fields] AND “reality”[All Fields]) OR “virtual reality”[All Fields])) OR ((“virtual”[All Fields] OR “virtuality”[All Fields] OR “virtualization”[All Fields] OR “virtualized”[All Fields] OR “virtualizing”[All Fields] OR “virtuals”[All Fields]) AND (“google”[All Fields] OR “google s”[All Fields] OR “googled”[All Fields] OR “googling”[All Fields]))

**Table 1:** Database search strategies.

Database	Search strategy	Results, n
PubMed	[“criteria” OR “standard”] AND [“develop” OR “recommend”] AND [“virtual reality” AND “google”]	109
Cochrane Library	criteria OR standard (select Record Title) AND develop OR recommend (select Abstract) AND virtual reality (select Record Title)	72
Embase	criteria OR standard AND develop OR recommend AND virtual reality AND google {related terms are included}	48
CINAHL	criteria OR standard (select TI Title) AND develop OR recommend (select AB Abstract) AND virtual reality AND google (select TI Title)	59
MEDLINE	criteria OR standard AND develop OR recommend AND virtual reality AND google	98
Scopus	criteria OR standard AND develop OR recommend AND virtual reality AND google (TITLE-ABS-KEY)	81
ZB MED	Criteria (open search) AND develop (open search AND virtual reality (title)	173
ACM DL	[[Title: criteria] OR [Title: standard]] AND [[Abstract: develop] OR [Abstract: recommend]] AND [[Title: virtual reality] AND [Title: google]]	121
IEEE Computer Society Digital Library	criteria OR standard (select Document Title) AND develop OR recommend (select Abstract) AND virtual reality AND google (select Document Title)	115

Publications were included in the following scenarios (inclusion criteria): (1) involved an examination (generally valid) of criteria for development recommendations for VR applications; (2) were focused on VR; (3) were written in English or German or both; (4) full-text articles were freely available; (5) involved studies were relevant to the subject (ie, focusing on older adults), and (6) had been published after 2012.

According to Hülsbömer [17], VR has seen a surge in popularity since 2012, leading to a greater emphasis on its development and ongoing discussions and optimizations of development processes. Given the rapid technological advancements and increased demands in the digital age, it is crucial to include relevant literature to ensure the relevance and validity of the core set of quality criteria [18].

In addition, publication types with the following designs were included: articles in scientific journals, books, book chapters, study reports, guidelines, assessment instruments/assessments, legislative regulations, and reports. As the goal was to provide a comprehensive initial overview of recommendations from the VR development literature, there were no restrictions on the type of study designs reviewed.

Exclusion criteria were as follows: (1) literature results not available in German or English; (2) results written before 2012; (3) study designs in the form of laboratory studies, case reports, and series; and (4) inappropriate publication types, such as book reviews, introductions, forewords, commentaries, position statements, and letters.

**Figure 2:**
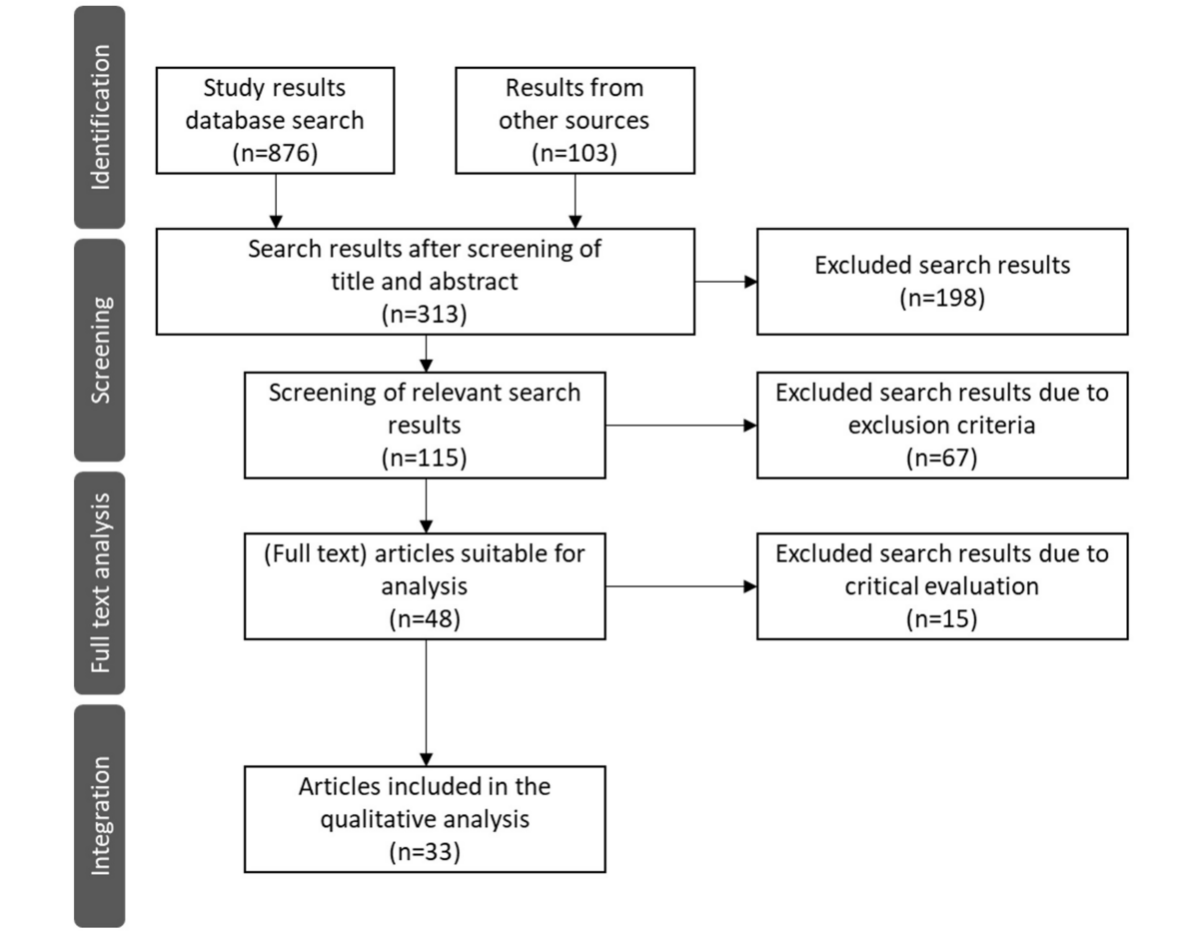
PRISMA (Preferred Reporting Items for Systematic Reviews and Meta-Analyses) flowchart of literature research procedure.

For searches in the databases (PubMed, Cochrane Library, Embase, CINAHL, MEDLINE, Scopus, ZB MED, IEEE Computer Society Digital Library, and ACM Digital Library), combinations of all 3 keywords were used, as exemplified in Textbox 1 for the search string used on PubMed. The systematic literature search, including the analysis process, was conducted between February 2021 and April 2021. Some of the publications found in the databases meeting the inclusion and exclusion criteria were partially identified as duplicates. All relevant studies identified by the database searches were downloaded and stored in the literature management software EndNote (Clarivate Plc), which automatically eliminated duplicates. Based on the search strategy, 876 results were identified from the database search and 103 results from other sources. The selection process is illustrated using a PRISMA (Preferred Reporting Items for Systematic Reviews and Meta-Analyses) flowchart (Figure 2). The procedure is comparable to that of Moher et al [19]. After screening the titles/abstracts, 313 publications remained, of which 198 studies were excluded, which mainly dealt with informatics-based guideline development and app development (eg, health apps or digital health apps for smartphones). Among the excluded articles, the full text was not freely available in 67. Thereafter, the remaining 115 publications were screened. Prefaces or introductory texts, statements, and general position papers were excluded at this point. Two independent reviewers assessed each title or abstract without influencing each other’s decisions. The reviewers first assessed the title/abstracts for eligibility, and in the next stage assessed the full text. To perform the screening process, category formation (inclusion or exclusion) was used with EndNote. A third reviewer determined final eligibility when a discrepancy existed between the initial reviewers. Thus, 48 articles were identified for the full-text analysis. In addition, 15 results were excluded because they were thematically unsuitable, focused on only 1 topic (eg, data privacy), and were therefore not comprehensive enough. In total, 33 studies were included based on the inclusion and exclusion criteria.

The literature search yielded enough freely available literature to establish the initial structure of the core quality criteria. The identified results provided valuable initial insights for the research project. Additionally, the workshop results play a crucial role in the participatory approach, potentially informing content specifics for future older adults. Hence, we deemed the approach suitable and ruled out potential bias. Multimedia Appendix 1 displays all publications included in this study for constructing the initial quality criteria core set [20-51].

### Framework Analysis

The content of the literature findings was analyzed using framework analysis, which involved iterative refinement of data-driven themes [52]. We chose framework analysis for its suitability in analyzing group-level qualitative data in research projects with specific goals, such as co-design. Framework analysis consists of 6 interrelated steps, which were conducted in this study as described in Textbox 2. The analysis was conducted by 2 researchers in Microsoft Excel (Microsoft Corporation). Based on the initial notes, a set of preliminary codes was developed for different aspects based on recurring themes from the literature. When disagreements arose regarding the codes, the 2 researchers discussed their conflicting interpretations and attempted to reach a consensus, possibly leading to the generation of new codes. The saturation of the data was determined by the 2 researchers. They jointly decided when saturation was reached for the formation of the first construct, which formed the basis for the expert workshops. The generation of additional themes and codes or categories and criteria took place in the 2 workshops.

**Framework analysis methodology, modified from Ritchie and Lewis [52].**

1. **Familiarization**
  This step involves a comprehensive familiarization with the data. For the development of the quality criteria core set, the individual results of the systematic literature review search (SLS) were reviewed to familiarize with the content. The initial content was developed by forming initial codes and themes. After all literature results had gone through this process, the data set was again fully analyzed.
2. **Identify recurring and important themes**
  The theoretical framework was developed by reviewing the selected results of the SLS and synthesizing the content deemed relevant to answer the research question. Data summaries were prepared to present the data in a concise format. In addition, an initial tabulation was created with preliminary codes that were grouped in the next steps.
3. **Indexing**
  In the indexing phase, the codes were grouped and classified into framework categories. The generated codes and themes were applied to the data summaries.
4. **Charting**
  The multilayered data were systematized and structured according to the theoretical framework. Codes were assigned to categories based on their content match. Initially, some content was extracted from the literature, in terms of both content assignment and its execution. The summaries of the data were reorganized under the generated themes of the theoretical framework and rewritten in a more abstract manner to reflect the themes.
5. **Analyzing**
  The generated data tables were analyzed and discussed by the 2 researchers for content and detail. The analysis included checking for content fit as well as comparability of granularity. Codes with comparable content were merged. In addition, whether contradictions existed between the codes were analyzed. Topic summaries were created to present the findings at a high level in the context of the research question.
6. **Interpretation**
  Interpretation included discussion of content and interpretation of individual categories or codes. It can be assumed that the interpretation leaves room for the development of further criteria that were not generated in the previous steps. Content was also evaluated for complexity and completeness. Descriptions and interpretations of the themes are presented later. Explanations and insights into the themes are considered in the “Discussion” section.

### Expert Workshops

The previously elaborated results of the 2 researchers were validated by conducting workshops and involving experts. Two workshops with different experts assessed the results for content fit, completeness, and level of granularity.

The first workshop, held in June 2021 due to COVID-19 pandemic restrictions, took place online and lasted for 2 hours. By involving 12 VR development experts, the previous results were revised using an interactive online collaboration platform. The experts were either self-employed or had over 10 years of experience in VR development research. The external professional reflection helped to refine and specify previous results. For the workshop, we used the MIRO platform [53] to map the previously developed results on digital boards.

The results of the framework analysis presented on the digital board were displayed on sliding digital sticky notes. The experts modified the existing structure by adding elaborations to existing criteria and generating new criteria or content using digital sticky notes. The content was generated by answering the guiding questions. The experts could freely modify the existing content of the template. In a joint discussion round, each content point was taken up, explained, and justified by the experts. In addition, the discussion aspects were recorded and an associated protocol of the respective workshops was used for the revision. The subsequent research steps involved editing the structure of the previous quality criteria core set by incorporating the results of the workshop. This was performed by assigning individual workshop contents, which were added to the categories or explanations of the criteria. New criteria were also generated. Accordingly, the content and structure of the previous version of the core set of quality criteria were adapted.

The second workshop included 12 VR application experts who had experience in the context of VR and older adults. The experts’ experience with VR and the target group of older adults was based, for example, on project experience or use in clinical settings (eg, hospitals or nursing homes). The consultation of VR application experts in this step aimed to bridge the gap between the prior findings and empirical observations gathered while working with older adults as part of the research process. The 2-hour online workshop took place in November 2021 and followed a procedure similar to that of the first workshop. The online collaboration platform was used again, and the results developed until this point were presented on the boards of the platform. The given categories or criteria were the same as those of the first workshop. The goal was to further refine and specify the results. The guiding questions were also identical to those of the first workshop to ensure the comparability of methods. The experts engaged in an interactive process to further refine the previous version of the quality criteria core set, followed by subsequent discussions to ensure its specificity and accuracy.

After the 2 workshops, the 2 researchers systematized the results by considering the protocol results of both workshops. The systematization of the results involved assessing the execution of content to ensure comparability in terms of the criteria’s level of detail and granularity. Changes were also made to the wording while preserving the original content’s meaning, ensuring a consistent structure. The outcome of this process serves as the foundation for VR application development and is referred to as the provisionally valid quality criteria core set.

### Ethical Considerations

The study was approved by the Ethics Committee of the German Sport University Cologne (Institute of Pedagogy and Philosophy; protocol number 095/22). As this is an observational study, no additional exemptions and approvals were necessary. Informed consent was given by all workshop participants. No secondary data analysis was performed. Participation in the workshop was voluntary. No personal data or data allowing conclusions to be drawn about the person were collected. All data were collected in an anonymized form. An ethics application was drafted and approved as part of adjacent activities of this research project. The submission of another, separate ethics application for the conduct of the workshops was waived. The Declaration of Helsinki also does not outline such a procedure for conducting a workshop in this particular format. Participants did not receive any compensation for taking part in the workshops.

## Results

### Systematic Literature Search

Following the completion of the SLS steps, which included identification, screening, full-text analysis, and integration, a total of 33 results were incorporated into the initial version of the quality criteria core set. The SLS uncovered the absence of a standardized set of quality criteria for VR applications, including those designed for older adults. To formulate the core set of quality criteria in this study, guidance from digital health applications (DiGA) in Germany was incorporated. These guidelines, such as AppQ and APPKRI, were selected because they have undergone multiple evaluations and are extensively described and assessed in terms of their content. Furthermore, it is worth noting that certain VR applications also qualify as DiGA, and as such, they were taken into account. The results of various studies provided valuable insights that were deemed essential for shaping the quality criteria core set. Additionally, specific legal provisions, such as those outlined in the Digital Health Care Act [54], were selectively integrated to ensure adherence to regulatory guidelines.

### Framework Analysis

The outcome of the framework analysis yielded an initial draft of the quality criteria core set, which encompassed a range of overarching categories and diverse criteria that addressed various content aspects relevant to older adult–friendly VR applications. Because of the differences in content, the development of the quality criteria core set was divided into 2 parts with the following 2 main topics: (1) “General Criteria for VR Application Design,” which included the general conditions/criteria of VR applications and (2) “Content Design of VR Applications,” which included criteria that are specifically attributed to the thematic content.

The results, that is, the framework analysis codes, framework analysis categories, and derived categories of the topic “General Criteria for VR Application Design,” are summarized in Table 2, and those for the topic “Content Design of VR Applications” are listed in Table 3. The framework analysis codes for the general criteria within the first category were *dependability, validity, objectivity, reliability, effectivity, knowledge, utility, treatment, improvement of (health) status, health, quality of life, health apps, psyche,* and *medical device.* As a result of this analysis, the derived framework analysis categories were *quality factors* and *medicine*. These findings led to the creation of the following categories within the first part: (1) medical quality, (2) data protection, (3) information security, (4) technical quality, (5) consumer protection and fairness, (6) interoperability, and (7) usability and motivation. The categories comprising the second part were as follows: (1) graphic/quality, (2) 3D character/avatar, (3) in-game instructions and prompts, (4) interaction, and (5) navigation.

The results of the framework analysis formed the basis for the subsequent workshops, in which the developed contents were discussed with the experts. All derived categories (ie, framework analysis categories) and framework analysis codes were disclosed in the expert workshops.

**Table 2:** Results of the framework analysis for the first category within the first part of the quality criteria core set focused on the “General Criteria for VR^a^ Application Design.”

Framework analysis codes (generated from literature)	Framework analysis category	Derived category
Dependability Validity Objectivity Reliability Effectivity Knowledge Utility	Quality factors	Medical quality
Treatment Improvement of (health) status Health Quality of life Health apps Psyche Medical device	Medicine	

^a^VR: virtual reality.

**Table 3:** Results of the framework analysis for the first category within the second part of the quality criteria core set focused on the “Content Design of VR^a^ Applications.”

Framework analysis codes (generated from literature)	Framework analysis category	Derived category
Software Degree of accuracy Structure Fineness/detail Concept Design	Graphical representation	Graphic/quality
VRISE (virtual reality–induced symptoms and effects) VR-Technology Texture Value Claim	Qualitative aspects	

^a^VR: virtual reality.

### Expert Workshops

Two expert workshops were conducted, drawing upon the data obtained from the systematic literature search and the framework analysis. In this process, a total of 7 categories were mapped to the first part, which encompassed “general quality criteria.” These categories were accompanied by example criteria derived from the literature. The second part involved “specific criteria” and was structured around 5 categories, each supported by example criteria sourced from the literature. The experts actively engaged in the process by collaboratively editing or adding content. The subsequent discussion was guided by the following key questions:

1. Are the categories (1-7, part 1 or 1-5, part 2) complete?

2. Which categories are missing/not appropriate for VR?

3. Are the criteria in each category comprehensive enough? Are criteria missing/can criteria be replaced?

The results of the first workshop with VR development experts are described in the following section.

The categories presented in Tables 2 and 3 were deemed appropriate by the experts, and there were no alterations or criticisms regarding their suitability. However, it is important to highlight that the extent and depth of input from participants varied across the 7 different categories. Notably, the categories “data protection” and “interoperability” received the fewest additional criteria. Conversely, the categories “technical quality” and “user-friendliness and motivation” saw the most substantial contributions. Nevertheless, it was necessary to scrutinize the results to identify and rectify any potential duplication, particularly in instances where the same criteria were described using different terms.

The results of the second workshop with VR application experts revealed the following: The given categories were not criticized or adapted by the application experts, and their suitability was found to be good. The input to the respective categories varied in terms of quantity and quality (ie, level of detail, execution, and description). The fewest criteria were added in the categories “information security” and “interoperability” (n=7 and n=4, respectively), whereas the highest number was added in the categories “technical quality” and “motivation” (n=19 and n=17, respectively). The results were checked for possible duplications. During the discussion, all categories and criteria were evaluated with the workshop participants. In this process, certain criteria were either retained or excluded for further development of the quality criteria core set. Most of these decisions on the respective contents were made unanimously among the participants. In instances where there were differing opinions, the decision-making process considered the majority agreement as the determining factor.

**Figure 3:**
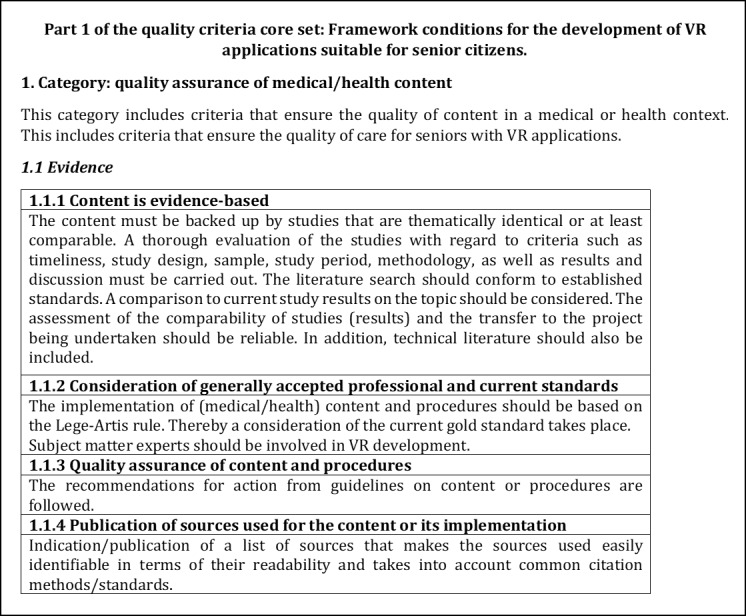
Excerpt of the first part of the quality criteria core set to the subcategory 1.1 Evidence.

**Figure 4:**
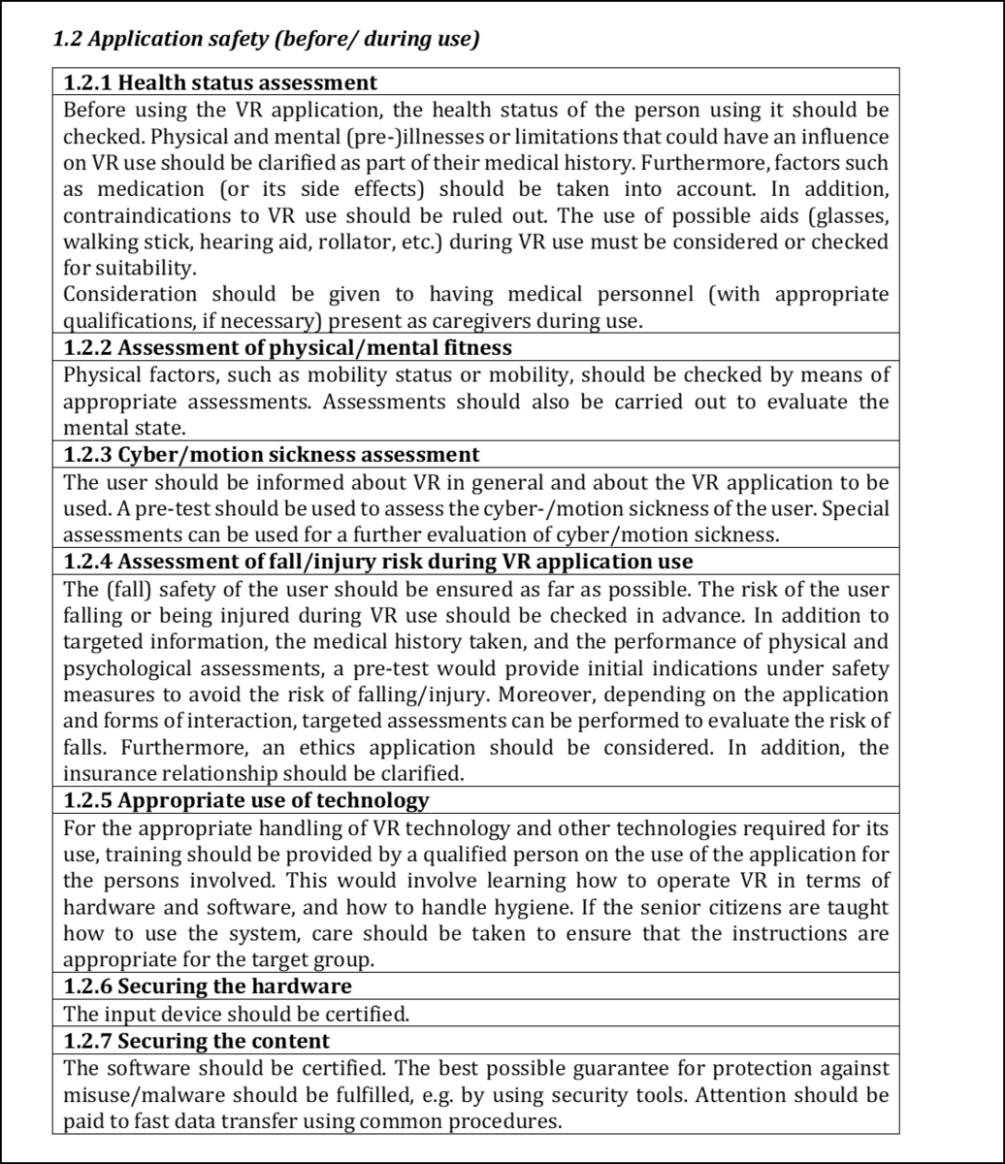
Excerpt of the first part of the quality criteria core set to the subcategory 1.2 Application safety (before/ during use).

**Figure 5:**
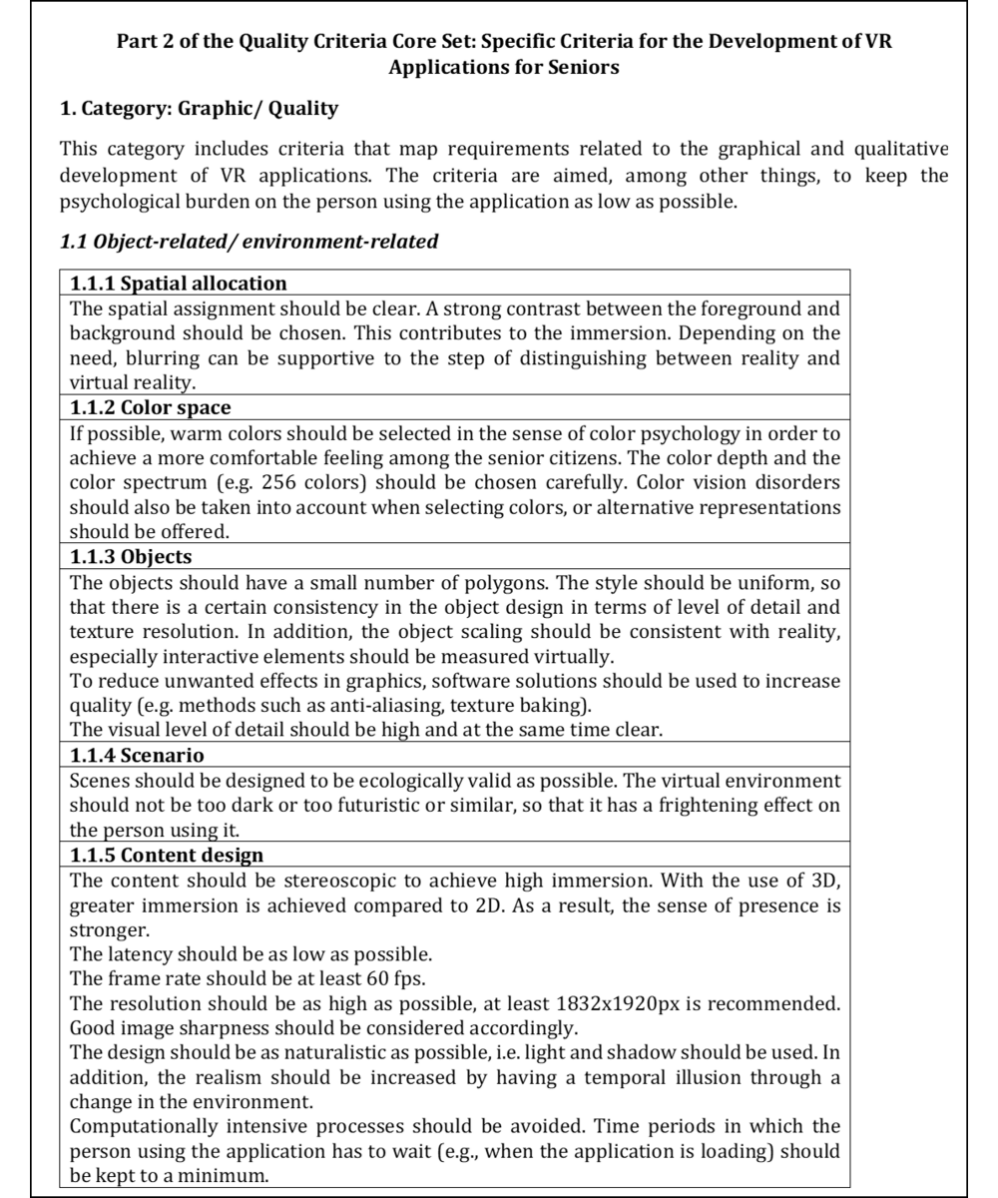
Excerpt of the second part of the quality criteria core set to the subcategory 1.1 Object related/ environment-related.

**Figure 6:**
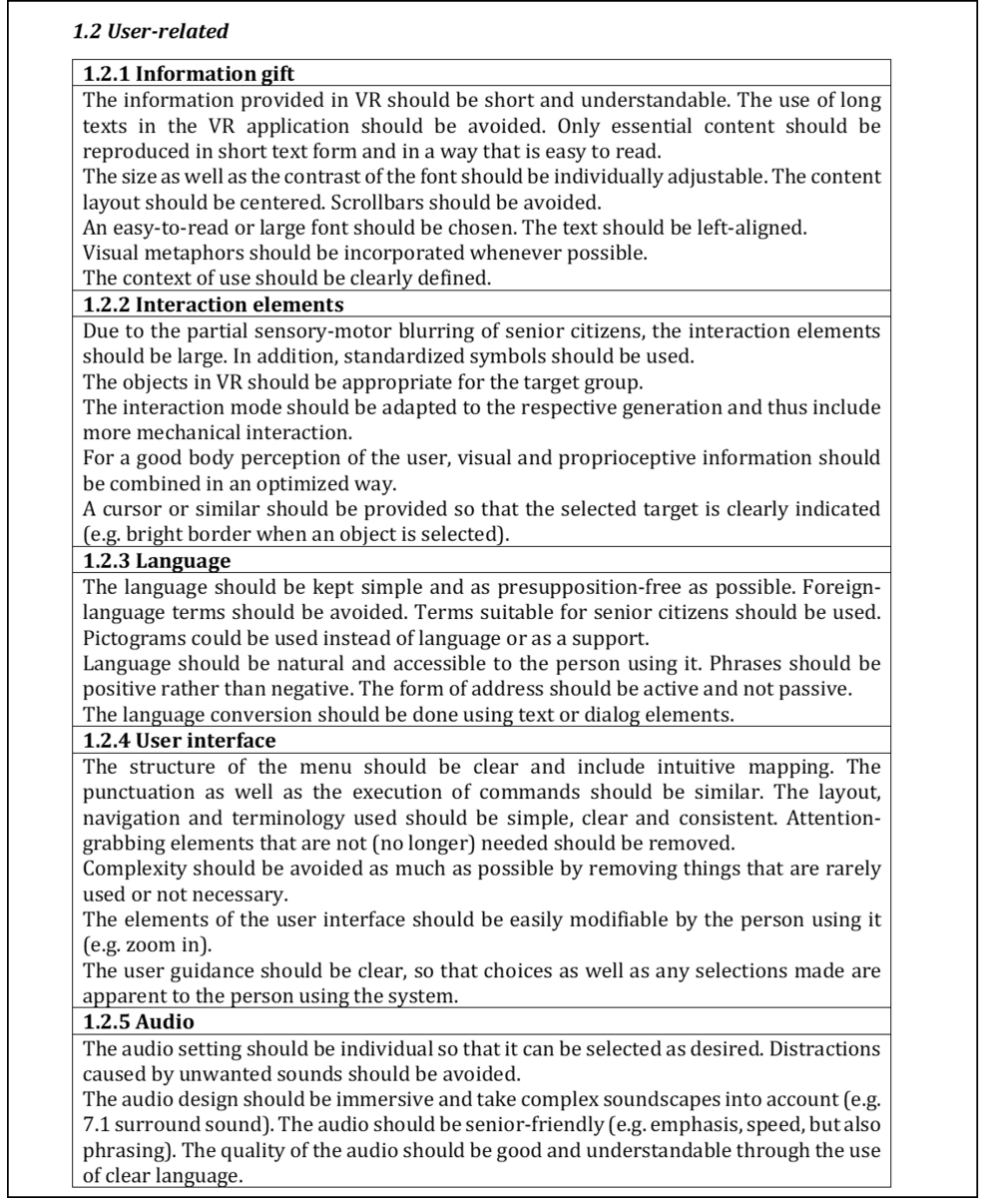
Excerpt of the second part of the quality criteria core set to the subcategory 1.2 User-related.

Following the discussion in the respective expert workshops, the results were revised or adjusted. This included the formation of subcategories as well as further criteria as a result of the reorganization of the content, which was based on the results of the workshops. The criteria were also further developed in terms of their content and categories were restructured. All changes made were based on the comments and opinions of the experts in both workshops. The current version of the quality criteria core set is briefly presented below. The first category of both parts is presented in detail in the respective subcategories (cf. Figures 3-6), whereas the remaining contents are presented in the form of categories and criteria (cf. Tables 4 and 5).

The content is derived from the codes or preliminary categories derived during the framework analysis. Based on thematic focus, subcategories were formed to differentiate the categories as well as to detail the criteria. Figure 3 shows a section of subcategory *1.1 Evidence,* which was assigned to the first category *Quality assurance of medical/health content* of the first part. The second subcategory of this part is *1.2 Application safety (before/during use)*, which is shown in Figure 4 with corresponding contents.

Figures 5 and 6 refer to the first category graphic/quality of the second part and depict corresponding criteria in the subcategories *1.1 Object-related/environment-related* and *1.2 User-related*, respectively, which were thematically subordinated to the subcategories.

The complete set of quality criteria with all versions can be requested from the authors.

**Table 4:** Presentation of categories, subcategories, and criteria of the first part of the quality criteria core set.

Category and subcategory		Criteria
**2. Data protection provisions**		
	2.1 Data generation and data storage	2.1.1 Earmarking 2.1.2 Necessity and data economy 2.1.3 Consent 2.1.4 Data security 2.1.5 Ensuring the authenticity of the data 2.1.6 Information security
	2.2 Legitimacies	2.2.1 Data protection law 2.2.2 Data participant rights 2.2.3 Accesses and access rights
**3. Quality requirements**		
	3.1 Technical safety	3.1.1 Robustness against disturbances 3.1.2 Hygiene
	3.2 User-related security	3.2.1 Avoiding the risk of collision 3.2.2 Suitable spectacle ergonomics 3.2.3 Change of operability 3.2.4 Traceability application situation
**4. Consumer protection**		
	4.1 Accessibility	4.1.1 User support 4.1.2 Conditions of use
	4.2 Transparency	4.2.1 Data/software update 4.2.2 Transparent business models
**5. Interoperability**		
	5.1 Data portability	5.1.1 Data extraction
	5.2 Technology compatibility	5.2 1 Connection of external technologies 5.2.2 Cross-generational use of technology

**Table 5:** Presentation of categories, subcategories, and criteria of the second part of the quality criteria core set.

Category and subcategory		Criteria
**2. 3D character/avatar**		
	2.1 Appearance	2.1.1 Character 2.1.2 Appearance/optics/optical representation
	2.2 Behaviorism	2.2.1 Behavior (facial expressions/gestures) 2.2.2 Tone and pitch of voice
**3. Providing in-game instructions and prompts**		
	3.1 Didactics	3.1.1 Initial learning 3.1.2 Instructions 3.1.3 Goal and task design 3.1.4 Feedback 3.1.5 Information transmission
	3.2 General conditions	3.2.1 Useful life 3.2.2 Local framework conditions
**4. Interaction**		
	4.1 Functionalities	4.1.1 Operation 4.1.2 Design/functionality 4.1.3 Relevance assessment
	4.2 Regulation	4.2.1 Evaluation/developments 4.2.2 Perception/proprioception
**5. Navigation**		
	5.1 Operation	5.1.1 Menu control 5.1.2 Control systems 5.1.3 Room design
	5.2 Taxis	5.2.1 Locomotion 5.2.2 Tracking
**6. Promotion of user motivation and loyalty to use**		
	6.1 Usability	6.1.1 Motivation 6.1.2 Logging 6.1.3 Configuration
	6.2 Target group orientation	6.2.1 Adaptability/customizability for different needs and interests 6.2.2 Practicability 6.2.3 User-friendliness

## Discussion

### Overview

The study’s objective was to establish a foundational set of quality criteria to provide guidance for the development of VR applications catering to older adults. The findings encompassed a diverse range of quality criteria derived from various qualitative research methods. This study represents the pioneering effort to create a core set of criteria specifically tailored to the development of VR applications for older adults. It introduced a novel qualitative methodological approach that involved a systematic review search, followed by framework analysis and validation through expert workshops, to formulate these criteria.

### Principal Findings

The results of the preliminary quality criteria core set provide an initial basis for older adult–friendly VR development. The individual criteria and categories are derived from the results of the SLS, the framework analysis, and the 2 expert workshops. It should be noted that the SLS research results included some of the German literature that was considered in the development process. This is attributed to the fact that the results of the quality criteria core set are primarily applicable to Germany and, as such, consider the specific regulations of the German health care system and legislation. Nevertheless, it is not precluded that these results can be extrapolated to other countries. In such instances, it is advisable to carefully examine the corresponding regulations that may diverge at the national or international level.

The validity of the criteria hinges on the research findings and the latitude for interpretation inherent in the reflective processes of the researchers involved. The expert workshops were thus crucial for the validation of the results. The results represent the criteria developed in the iterative process, which are detailed using explanation examples. Considering the thorough survey conducted across various databases and the consensus among experts during the 2 workshops, it is reasonable to consider the resulting quality criteria core set as a typical representation of a preliminary version. However, the correctness or durability of the results is uncertain. Given the rapid development of technology, particularly in the VR market, it must be assumed that the criteria within the provisional core set of quality criteria are only valid to a limited extent. Nevertheless, this version forms a first building block and serves as a guideline for basic VR development for the target group of older adults.

The categorization of criteria into their respective subcategories (as seen in Figures 3-6) is guided by thematic considerations. However, it is important to acknowledge that thematic overlaps may occur, and the precise classification of criteria into specific subcategories may need to be reevaluated based on the context of the application. For the initial draft of the quality criteria core set, which serves as the foundation, the current structuring within the subcategories and sorting is deemed appropriate.

The distribution of criteria among the respective (sub)categories is somewhat uneven, with varying numbers of criteria in each category. This suggests that certain topic areas receive more extensive coverage than others. An illustrative example of this is the category of data protection, which encompasses a broader range of content within the quality criteria core set compared with other categories. One plausible explanation for this discrepancy is that certain topic areas are subject to stringent standards and requirements, resulting in a greater abundance of detailed explanations compared with other categories.

For a general understanding, these criteria are explained in detail. However, the degree of granularity must be considered: The understanding and interpretation of the explanations vary depending on the individual’s level of knowledge, experience, and assessment. Therefore, explanations provided for the individual criteria leave some room for interpretation. The fulfillment of the criteria depends on the respective framework conditions and the hardware. VR head-mounted displays and other VR hardware have different functionalities. Therefore, the requirements developed in the quality criteria form may not be fulfilled or only be insufficiently fulfilled in some cases. The functionalities of the VR hardware also determine the definition and fulfillment of the quality criteria.

The results should reflect older adult–friendly criteria. However, the heterogeneity of the target group must be considered. In old age, numerous physiological and psychological changes occur within individuals. Accordingly, the requirements for VR applications or VR systems may vary within the target group of older adults [55]. Defining older adults by age could assist in refining the criteria, while also considering the stereotypical characteristics associated with each age group. Older adults may use VR in different contexts [56]. To specify the criteria for VR applications suitable for older adults, a classification of application areas could also be helpful. Moreover, the purpose of the VR application is significant, as are considerations about the motivations behind VR application development. The purpose of VR needs to be thoroughly justified. Additionally, older adults’ level of technology familiarity and willingness to use VR technology influence these considerations [57,58]. The development of VR applications for older adults requires an analysis of the necessary functionalities relevant to the development project in the initial conceptual phase. The preliminary valid core set of quality criteria serves as a guideline to specify and define further aspects after initial considerations for application development. The validity of the individual criteria must be determined individually and possibly modified depending on the application purpose. In principle, these criteria can serve as valuable guidelines to facilitate more focused design development, beginning from the conceptual stage. By considering these criteria throughout subsequent phases, informed decisions can be made regarding their relevance and integration into the design process.

The significance of quality criteria is continually increasing, and it has become an important topic for discussion. Manser and de Bruin [59] have also called for quality criteria that are thematically related to exergames and described a different approach; however, their study also focused on the target group of older adults [59]. The authors refer to the framework proposed by Li et al [60], which represents a comparable methodological approach to this work but does not clearly map defined quality criteria.

It should be noted that for the development of a targeted VR application for older adults, a participatory approach is important. In addition to people from the creative industry (eg, VR developers, game designers, computer scientists) and people from the relevant application area, the target group should be included. The existing version of the core set of quality criteria for older adult–friendly VR applications will undergo further refinement through the inclusion of older adults in future research projects. The VR development work should thus occur through collaborative and transparent exchanges with both current users and prospective users. Feedback should also be evaluated and taken into account with regard to technical feasibility. In pretest phases and based on prototypes, new insights can be gained that can be used to further specify the core set of quality criteria.

### Limitations

Individual categories or criteria, such as “data protection,” must be dealt with more comprehensively to ensure the validity of the content. This example category often shows significant changes, as the topic is currently undergoing constant revision and also needs to be adapted to country-specific requirements.

The theoretical construct in the form of the preliminary valid core set of quality criteria will be tested in the next step involving practical implementation. The criteria developed so far will be incorporated into the development work so that practical testing can determine whether the individual criteria can be met. In an upcoming workshop with older adults on the current version of the quality criteria core set, the criteria will be discussed and put into their final form. When discussion and refinement of the content is complete, the version is referred to as the “evaluated and standardized quality criteria core set.” Thereafter, the updated results will be presented in a follow-up publication, in which the target group of older adults will be directly included and verification of the theoretically constructed quality criteria will be performed using a VR application in practice.

### Conclusions

User-centered development is useful if a product or measure is to reach the target audience. The quality criteria core set should act as a kind of guideline for the implementation of the targeted development work. Attention should be paid to a possible specification of the user groups and the context of use. In addition, the heterogeneity of the target group of older adults should be considered. The quality criteria core set serves as an initial step toward user-centered VR application development, but additional research is needed to build upon the existing results and further enhance the core set.

## Appendix

Multimedia Appendix 1 Included publications according to systematic literature review search (SLS). See also [20-51].

## Abbreviations

DiGA: digital health applications
PRISMA: Preferred Reporting Items for Systematic Reviews and Meta-Analyses
SLS: systematic literature review search
VR: virtual reality
WHO: World Health Organization

## Data Availability

The data sets are provided in the main manuscript by the authors.

## References

[1] Cipresso P, Giglioli IAC, Raya MA, Riva G (2018). The past, present, and future of virtual and augmented reality research: a network and cluster analysis of the literature. Front Psychol.

[2] Dermody G, Whitehead L, Wilson G, Glass C (2020). The role of virtual reality in improving health outcomes for community-dwelling older adults: systematic review. J Med Internet Res.

[3] Freeman D, Reeve S, Robinson A, Ehlers A, Clark D, Spanlang B, Slater M (2017). Virtual reality in the assessment, understanding, and treatment of mental health disorders. Psychol Med.

[4] Liao Y, Tseng H, Lin Y, Wang C, Hsu W (2020). Using virtual reality-based training to improve cognitive function, instrumental activities of daily living and neural efficiency in older adults with mild cognitive impairment. Eur J Phys Rehabil Med.

[5] Maggio MG, Latella D, Maresca G, Sciarrone F, Manuli A, Naro A, De Luca R, Calabrò Rocco Salvatore (2019). Virtual reality and cognitive rehabilitation in people with stroke: an overview. J Neurosci Nurs.

[6] Faria AL, Andrade A, Soares L, I Badia SB (2016). Benefits of virtual reality based cognitive rehabilitation through simulated activities of daily living: a randomized controlled trial with stroke patients. J Neuroeng Rehabil.

[7] Ortet CP, Veloso AI, Vale Costa L (2022). Cycling through 360° virtual reality tourism for senior citizens: empirical analysis of an assistive technology. Sensors (Basel).

[8] Tobler-Ammann BC, Surer E, Knols RH, Borghese NA, de Bruin Eling D (2017). User perspectives on exergames designed to explore the hemineglected space for stroke patients with visuospatial neglect: usability study. JMIR Serious Games.

[9] Aydin S, Aktaş B (2020). Developing an integrated VR infrastructure in architectural design education. Front Robot AI.

[10] Kyaw BM, Saxena N, Posadzki P, Vseteckova J, Nikolaou CK, George PP, Divakar U, Masiello I, Kononowicz AA, Zary N, Tudor Car L (2019). Virtual reality for health professions education: systematic review and meta-analysis by the digital health education collaboration. J Med Internet Res.

[11] Chen P, Krch D (2022). Immersive virtual reality treatment for spatial neglect: an agile, user-centered development process. Ann Phys Rehabil Med.

[12] Rowe JW, Kahn RL (1987). Human aging: usual and successful. Science.

[13] Rudnicka E, Napierała P, Podfigurna A, Męczekalski B, Smolarczyk R, Grymowicz M (2020). The World Health Organization (WHO) approach to healthy ageing. Maturitas.

[14] Dziechciaż M, Filip R (2014). Biological psychological and social determinants of old age: bio-psycho-social aspects of human aging. Ann Agric Environ Med.

[15] Afifi T, Collins NL, Rand K, Fujiwara K, Mazur A, Otmar C, Dunbar NE, Harrison K, Logsdon R (2021). Testing the feasibility of virtual reality with older adults with cognitive impairments and their family members who live at a distance. Innov Aging.

[16] Birckhead B, Khalil C, Liu X, Conovitz S, Rizzo A, Danovitch I, Bullock K, Spiegel B (2019). Recommendations for methodology of virtual reality clinical trials in health care by an international working group: iterative study. JMIR Ment Health.

[17] Hülsbömer S (2015). Hype Cycles der letzten zehn Jahre: Gartner-Trends im Reality Check. COMPUTERWOCHE.

[18] Rollwagen I (2015). Zeit und Innovation: Zur Synchronisation von Wirtschaft, Wissenschaft und Politik bei der Genese der Virtual-Reality-Technologien.

[19] Moher D, Liberati Alessandro, Tetzlaff Jennifer, Altman Douglas G, PRISMA Group (2009). Preferred reporting items for systematic reviews and meta-analyses: the PRISMA statement. Ann Intern Med.

[20] Fraunhofer-Institut für Offene Kommunikationssysteme (2018). APPKRI Kriterien für Gesundheits-Apps. Fraunhofer-Institut für Offene Kommunikationssysteme [FOKUS].

[21] Bertelsmann Stiftung (2019). AppQ: Gütekriterien-Kernset für mehr Qualitätstransparenz bei digitalen Gesundheitsanwendungen. Bertelsmann Stiftung.

[22] Stoyanov Stoyan R, Hides Leanne, Kavanagh David J, Zelenko Oksana, Tjondronegoro Dian, Mani Madhavan (2015). Mobile app rating scale: a new tool for assessing the quality of health mobile apps. JMIR Mhealth Uhealth.

[23] Baumel Amit, Faber Keren, Mathur Nandita, Kane John M, Muench Fred (2017). Enlight: a comprehensive quality and therapeutic potential evaluation tool for mobile and web-based eHealth interventions. J Med Internet Res.

[24] Haute Autorité de Santé (HAS) (2016). Good practice guidelines on health apps and smart devices (mobile health or mhealth). Haute Autorité de Santé (HAS).

[25] National Health Service Digital (2018). Digital Assessment Questionnaire V2.1. National Health Service Digital.

[26] American Psychiatric Association (APA) (2018). The App Evaluation Model. American Psychiatric Association (APA).

[27] Bertelsmann Stiftung (2020). AppQ 1.1: Gütekriterien-Kernset für mehr Qualitätstransparenz bei digitalen Gesundheitsanwendungen. Bertelsmann Stiftung.

[28] Bundesinstitut für Arzneimittel und Medizinprodukte (BfArM) (2020). DiGA-Leitfaden. Bundesinstitut für Arzneimittel und Medizinprodukte (BfArM).

[29] Bundesinstituts für Arzneimittel und Medizinprodukte (BfArM) (2016). BfArM-Orientierungshilfe medical apps. Bundesinstituts für Arzneimittel und Medizinprodukte (BfArM).

[30] Bundesministerium für Gesundheit (BMG) (2020). Digitale-Gesundheitsanwendungen-Verordnung (DiGAV). Bundesministerium für Gesundheit (BMG).

[31] Bertelsmann Stiftung (2016). Digital-Health-Anwendungen für Bürger: Kontext, Typologie und Relevanz aus Public-Health-Perspektive. Bertelsmann Stiftung.

[32] Bertelsmann Stiftung (2019). Transfer von Digital-Health-Anwendungen in den Versorgungsalltag (Teil 6), Teil 6: Transparenzmodell Digital-Health-Anwendungen – Grundlagen, Herleitung und Modell. Bertelsmann Stiftung.

[33] Albrecht UV (2016). Chancen und Risiken von Gesundheits-Apps (CHARISMHA). Technische Universität Braunschweig.

[34] Bucksch M (2021). Leitfaden für die Entwicklung von Medical Apps: Darauf müssen Hersteller achten. QuickBird Medical.

[35] Aktionsforum Gesundheitsinformationssystem (afgis) (2012). Gesundheits-App Fact Sheet. Aktionsforum Gesundheitsinformationssystem (afgis).

[36] Aktionsforum Gesundheitsinformationssystem (afgis) (2021). afgis-Transparenzkriterien. Aktionsforum Gesundheitsinformationssystem (afgis).

[37] Universitätsklinikum Freiburg (2013). Gesundheits- und Versorgungs-Apps. Universitätsklinikum Freiburg.

[38] Europäische Kommission (2014). Grünbuch über Mobile-Health-Dienste (mHealth). Bundesrat.

[39] Bruder R, Eckert T, Conradt J, Caserman P, Schaub M, Hofmann K, Wiemeyer J, Straßburg K, Müller P, Göbel S (2021). Gütekriterien Serious Games - Langfassung 30.03.2021. TU Darmstadt.

[40] Kourtesis P, Korre D, Collina S, Doumas Laa, MacPherson Se (2020). Guidelines for the development of immersive virtual reality software for cognitive neuroscience and neuropsychology: the development of Virtual Reality Everyday Assessment Lab (VR-EAL), a neuropsychological test battery in immersive virtual reality. Front Comput Sci.

[41] Cong Xu, Li Tingting (2020). Design and development of virtual medical system interface based on VR-AR hybrid technology. Comput Math Methods Med.

[42] Vogel J, Schuir J, Thomas O, Teuteberg F (2020). Gestaltung und erprobung einer virtual-reality-anwendung zur unterstützung des prototypings in design-thinking-prozessen. HMD.

[43] Kourtesis Panagiotis, Collina Simona, Doumas Leonidas A A, MacPherson Sarah E (2019). Technological competence is a pre-condition for effective implementation of virtual reality head mounted displays in human neuroscience: a technological review and meta-analysis. Front Hum Neurosci.

[44] Madary M, Metzinger Tk (2016). Recommendations for good scientific practice and the consumers of VR-technology. Front Robot AI.

[45] Boletsis C, Cedergren Je (2019). VR locomotion in the new era of virtual reality: an empirical comparison of prevalent techniques. Advances in Human-Computer Interaction.

[46] LaValle SM (2015). Virtual Reality.

[47] Govea-Valladares Eder H, Medellin-Castillo Hugo I, Ballesteros Jorge, Rodriguez-Florido Miguel A (2018). On the development of virtual reality scenarios for computer-assisted biomedical applications. J Healthc Eng.

[48] Bundesverband Informationswirtschaft, Telekommunikation und neue Medien (Bitkom) e.V (2021). Augmented und Virtual Reality: Potenziale und praktische Anwendung immersiver Technologien. Bitkom.

[49] Brennesholtz MS (2018). 3‐1: Invited Paper: VR standards and guidelines. SID Symposium Digest of Technical Papers 49.

[50] Stach M, Kraft R, Probst T, Messner EM, Terhorst Y, Baumeister H, Schickler M, Reichert M, Sander LS, Pryss R (2020). Mobile health app database - a repository for quality ratings of mHealth apps.

[51] Yuan Y (2018). Paving the road for virtual and augmented reality [standards]. IEEE Consumer Electron Mag.

[52] Ritchie J, Lewis J (2003). Qualitative Research Practice: A Guide for Social Science Students and Researchers.

[53] MIRO.

[54] (2009). Gesetz für eine bessere Versorgung durch Digitalisierung und Innovation (Digitale-Versorgung-Gesetz - DVG). Bundesgesetzblatt.

[55] Garcia JA (2019). A virtual reality game-like tool for assessing the risk of falling in the elderly. Stud Health Technol Inform.

[56] Perez-Marcos D (2018). Virtual reality experiences, embodiment, videogames and their dimensions in neurorehabilitation. J Neuroeng Rehabil.

[57] Cook N, Winkler SL (2016). Acceptance, usability and health applications of virtual worlds by older adults: a feasibility study. JMIR Res Protoc.

[58] Roccetti M, Prandi C, Mirri S, Salomoni P (2020). Designing human-centric software artifacts with future users: a case study. Hum Cent Comput Inf Sci.

[59] Manser P, de Bruin ED (2021). Making the best out of IT: design and development of exergames for older adults with mild neurocognitive disorder - a methodological paper. Front Aging Neurosci.

[60] Li Y, Muñoz J, Mehrabi S, Middleton L, Cao S, Boger J, Fang X (2020). Multidisciplinary iterative design of exergames (MIDE): a framework for supporting the design, development, and evaluation of exergames for health. HCI in Games.

