# Supplementary material for: Development of a Core Set of Quality Criteria for Virtual Reality Applications Designed for Older Adults: Multistep Qualitative Study
Source: Interact J Med Res. 2023 Sep 27;12:e45433. doi: 10.2196/45433 (PMC10568390; doi:10.2196/45433)
Supplement: Multimedia Appendix 1 [file ijmr_v12i1e45433_app1.docx]

|  | Titel | Publication |
| --- | --- | --- |
| 1 | AppQ 0.9  Gütekriterien-Kernset für mehr Qualitätstransparenz bei digitalen Gesundheitsanwendungen | Bertelsmann Stiftung (2019) [1] |
| 2 | APPKRI Kriterien für Gesundheits-Apps | Fraunhofer-Institut für Offene Kommunikationssysteme (2018) [2] |
| 3 | AppQ 1.0  Gütekriterien-Kernset für mehr Qualitätstransparenz bei digitalen Gesundheitsanwendungen | Bertelsmann Stiftung (2019) [1] |
| 4 | Mobile Application Rating Scale (MARS) | Stoyanov et al., (2015) [3] |
| 5 | ENLIGHT | Baumel et al. (2017) [4] |
| 6 | Good Practice Guidelines on Health Apps and Smart Devices | Haute Autorité de Santé (2016) [5] |
| 7 | Digital Assessment Questions Beta | National Health Service Digital (2018) [6] |
| 8 | App Evaluation Model | American Psychiatric Association (2018) [7] |
| 9 | AppQ 1.1  Gütekriterien-Kernset für mehr Qualitätstransparenz bei digitalen Gesundheitsanwendungen | Bertelsmann Stiftung (2020) [8] |
| 10 | DiGA-Leitfaden: Das Fast Track Verfahren für digitale Gesundheitsanwendungen (DiGA) nach § 139e SGB V – Ein Leitfaden für Hersteller, Leistungserbringer und Anwender | Bundesinstituts für Arzneimittel und Medizinprodukte (2020) [9] |
| 11 | BfArM-Orientierungshilfe Medical Apps | Bundesinstituts für Arzneimittel und Medizinprodukte (2016) [10] |
| 12 | Digitale Gesundheitsanwendungen-Verordnung - DiGAV | Bundesministerium für Gesundheit (2020) [11] |
| 13 | Digital-Health-Anwendungen für Bürger  Kontext, Typologie und Relevanz aus Public-Health-Perspektive | Bertelsmann Stiftung (2016) [12] |
| 14 | Transfer von Digital-Health-Anwendungen in den Versorgungsalltag (Part6)  Part6: Transparenzmodell Digital-Health-Anwendungen – Grundlagen, Herleitung und Modell | Bertelsmann Stiftung (2019) [13] |
| 15 | CHARISMHA - Chancen und Risiken von Gesundheits-Apps | Albrecht, U.-V. (2016) [14] |
| 16 | Leitfaden für die Entwicklung von Medical Apps: Darauf müssen Hersteller achten | Bucksch, M. (2021) [15] |
| 17 | Gesundheits-App Fact Sheet | Aktionsforum Gesundheitsinformationssystem (2012) [16] |
| 18 | afgis-Transparenzkriterien | Aktionsforum Gesundheitsinformationssystem (2021) [17] |
| 19 | Gesundheits- und Versorgungs-Apps: Hintergründe zu deren Entwicklung und Einsatz | Universitätsklinikum Freiburg (2013) [18] |
| 20 | GRÜNBUCH über Mobile-Health-Dienste („mHealth“) | Europäische Kommission (2014) [19] |
| 21 | Gütekriterien Serious Games-Langfassung | Bruder et al. (2021) [20] |
| 22 | Guidelines for the Development of Immersive Virtual Reality Software for Cognitive Neuroscience and Neuropsychology: The Development of Virtual Reality Everyday Assessment Lab (VR-EAL), a Neuropsychological Test Battery in Immersive Virtual Reality | Kourtesis et al. (2020) [21] |
| 23 | Design and Development of Virtual Medical System Interface Based on VR-AR Hybrid Technology | Cong & Li (2020) [22] |
| 24 | Gestaltung und Erprobung einer Virtual-Reality-Anwendung zur Unterstützung des Prototypings in Design-Thinking-Prozessen. | Vogel et al. (2020) [23] |
| 25 | Technological Competence Is a Pre-condition for Effective Implementation of Virtual Reality Head Mounted Displays in Human Neuroscience: A Technological Review and Meta-Analysis. | Kourtesis et al. (2019) [24] |
| 26 | Real Virtuality: A Code of Ethical Conduct. Recommendations for Good Scientific Practice and the Consumers of VR-Technology | Madary & Metzinger (2016) [25] |
| 27 | VR Locomotion in the New Era of Virtual Reality: An Empirical Comparison of Prevalent Techniques | Boletsis & Cedergren (2019) [26] |
| 28 | Virtual Reality | LaValle S.M. (2015) [27] |
| 29 | On the Development of Virtual Reality Scenarios for Computer-Assisted Biomedical Applications | Govea-Valladares et al. (2018) [28] |
| 30 | Augmented und Virtual Reality: Potenziale und praktische Anwendung immersiver Technologien | Bitkom (2021) [29] |
| 31 | 3-1: Invited Paper: VR Standards and Guidelines | Brennesholtz, M. S. (2018) [30] |
| 32 | Mobile Health App Database - A Repository for Quality Ratings of mHealth Apps | Stach et al. (2021) [31] |
| 33 | Paving the Road for Virtual and Augmented Reality [Standards] | Yuan, Y. (2018) [32] |

## References

[1] Bertelsmann Stiftung. AppQ: Gütekriterien-Kernset für mehr Qualitätstransparenz bei digitalen Gesundheitsanwendungen. 2019. <https://www.bertelsmann-stiftung.de/de/publikationen/publikation/did/appq/> [accessed Oct 21, 2021].

[2] Fraunhofer-Institut für Offene Kommunikationssysteme [FOKUS]. APPKRI Kriterien für Gesundheits-Apps. 2018. <https://ehealth-services.fokus.fraunhofer.de/BMG-APPS> [accessed Oct 21, 2021].

[3] Stoyanov SR, Hides L, Kavanagh DJ, Zelenko O, Tjondronegoro D, Mani M (2015) Mobile App Rating Scale: A New Tool for Assessing the Quality of Health Mobile Apps. JMIR Mhealth Uhealth 2015;3(1):e27. doi: [10.2196/mhealth.3422](https://doi.org/10.2196/mhealth.3422)PMID: [25760773](https://www.ncbi.nlm.nih.gov/pubmed/25760773)PMCID: [4376132](https://www.ncbi.nlm.nih.gov/pmc/articles/4376132)

[4] Baumel A, Faber K, Mathur N, Kane JM, Muench F (2017) Enlight: A Comprehensive Quality and Therapeutic Potential Evaluation Tool for Mobile and Web-Based eHealth Interventions. J Med Internet Res 2017;19(3):e82. doi: [10.2196/jmir.7270](https://doi.org/10.2196/jmir.7270)PMID: [28325712](https://www.ncbi.nlm.nih.gov/pubmed/28325712)PMCID: [5380814](https://www.ncbi.nlm.nih.gov/pmc/articles/5380814).

[5] Haute Autorité de Santé [HAS]. Good practice guidelines on health apps and smart devices (mobile health or mhealth). Saint-Denis La Plaine. 2016. <https://www.has-sante.fr/jcms/c_2681915/en/good-practice-guidelines-on-health-apps-and-smart-devices-mobile-health-or-mhealth> [accessed Oct 21, 2021].

[6] National Health Service Digital (2018). Digital Assessment Questionnaire V2.1. 2018. <https://digital.nhs.uk/search?query=digital+assessment+questionnaire> [accessed Oct 21, 2021].

[7] American Psychiatric Association [APA]. The App Evaluation Model. 2018. <https://www.psychiatry.org/psychiatrists/practice/mental-health-apps/the-app-evaluation-model> [accessed Oct 21, 2021].

[8] Bertelsmann Stiftung. AppQ 1.1: Gütekriterien-Kernset für mehr Qualitätstransparenz bei digitalen Gesundheitsanwendungen. 2020. <https://www.bertelsmann-stiftung.de/de/publikationen/publikation/did/appq-1-1> [accessed Oct 21, 2021].

[9] Bundesinstitut für Arzneimittel und Medizinprodukte [BfArM] (2020). DiGA-Leitfaden. 2020. <https://www.bfarm.de/SharedDocs/Downloads/DE/Service/Beratungsverfahren/DiGA-Leitfaden.html> [accessed Oct 21, 2021].

[10] Bundesinstitut für Arzneimittel und Medizinprodukte [BfArM]. BfArM-Orientierungshilfe Medical Apps. 2016. <https://www.bfarm.de/SiteGlobals/Forms/Suche/Servicesuche_Formular.html;jsessionid=EB5D9FFA612077A546DEAD32D188F243.intranet262?nn=468782&resourceId=468548&input_=494988&pageLocale=de&templateQueryString=BfArM-Orientierungshilfe+Medical+Apps&submit.x=0&submit.y=0> [accessed Oct 21, 2021].

[11] Bundesministerium für Gesundheit [BMG]. Digitale-Gesundheitsanwendungen-Verordnung (DiGAV). 2020. <https://www.bundesgesundheitsministerium.de/service/gesetze-und-verordnungen/guv-19-lp/digav.html> [accessed Oct 21, 2021].

[12] Bertelsmann Stiftung. Digital-Health-Anwendungen für Bürger: Kontext, Typologie und Relevanz aus Public-Health-Perspektive. 2016. <https://www.bertelsmann-stiftung.de/de/publikationen/publikation/did/digital-health-anwendungen-fuer-buerger/> [accessed Oct 21, 2021].

[13] Bertelsmann Stiftung. Transfer von Digital-Health-Anwendungen in den Versorgungsalltag (Teil 6), Teil 6: Transparenzmodell Digital-Health-Anwendungen – Grundlagen, Herleitung und Modell. 2019. <https://www.bertelsmann-stiftung.de/de/publikationen/publikation/did/transfer-von-digital-health-anwendungen-in-den-versorgungsalltag-teil-6> [accessed Oct 21, 2021].

[14] Albrecht, U.-V. Chancen und Risiken von Gesundheits-Apps (CHARISMHA). 2016. <https://publikationsserver.tu-braunschweig.de/receive/dbbs_mods_00060000> [accessed Sep 22, 2020].

[15] Bucksch, M. Leitfaden für die Entwicklung von Medical Apps: Darauf müssen Hersteller achten. 2021. <https://quickbirdmedical.com/medical-app-entwicklung-mdr/> [accessed Oct 21, 2021].

[16] Aktionsforum Gesundheitsinformationssystem [afgis]. Gesundheits-App Fact Sheet. 2012. <https://www.afgis.de/standards/gesundheitsapps/> [accessed Oct 21, 2021].

[17] Aktionsforum Gesundheitsinformationssystem [afgis]. afgis-Transparenzkriterien. 2021. <https://www.afgis.de/zertifizierung/transparenzkriterien> [accessed Oct 21, 2021].

[18] Universitätsklinikum Freiburg. Gesundheits- und Versorgungs-Apps. 2013. <https://www.uniklinik-freiburg.de/fileadmin/mediapool/09_zentren/studienzentrum/pdf/Studien/150331_TK-Gesamtbericht_Gesundheits-und_Versorgungs-Apps.pdf> [accessed Oct 21, 2021].

[19] Europäische Kommission. Grünbuch über Mobile-Health-Dienste (mHealth). 2014. <https://www.bundesrat.de/SharedDocs/beratungsvorgaenge/2014/0101-0200/0167-14.html> [accessed Oct 21, 2021].

[20] Bruder, R., Eckert, T., Conradt, J., Caserman, P., Schaub, M., Hofmann, K. et al. Gütekriterien Serious Games – Langfassung 30.03.2021. 2021. <https://tuprints.ulb.tu-darmstadt.de/17872/> [accessed Oct 21, 2021].

[21] Kourtesis P, Korre D, Collina S, Doumas L and MacPherson S. Guidelines for the Development of Immersive Virtual Reality Software for Cognitive Neuroscience and Neuropsychology: The Development of Virtual Reality Everyday Assessment Lab (VR-EAL), a Neuropsychological Test Battery in Immersive Virtual Reality. Front. Comput. Sci. 1,12. 2020 Jan 14. doi: 10.3389/fcomp.2019.00012.

[22] Cong X, Li T. Design and Development of Virtual Medical System Interface Based on VR-AR Hybrid Technology. Comput Math Methods Med. 2020;2020:7108147. 2020 Aug 25. doi:10.1155/2020/7108147.

[23] Vogel J, Schuir J, Thomas O and Teuteberg F. Gestaltung und Erprobung einer Virtual-Reality-Anwendung zur Unterstützung des Prototypings in Design-Thinking-Prozessen. HMD 57, 432–450. 2020 Mar 26. doi: 10.1365/s40702-020-00608-9.

[24] Kourtesis P, Collina S, Doumas LAA, MacPherson SE. Technological Competence Is a Pre-condition for Effective Implementation of Virtual Reality Head Mounted Displays in Human Neuroscience: A Technological Review and Meta-Analysis. Front Hum Neurosci. 2019;13:342. 2019 Oct 2. doi:10.3389/fnhum.2019.00342.

[25] Madary M and Metzinger T. Real Virtuality: A Code of Ethical Conduct. Recommendations for Good Scientific Practice and the Consumers of VR-Technology. Front. Robot. AI, 16(3), 1-23. 2016 Feb 19. doi: 10.3389/frobt.2016.00003.

[26] Boletsis C and Cedergren J. VR Locomotion in the New Era of Virtual Reality: An Empirical Comparison of Prevalent Techniques, Advances in Human-Computer Interaction, 2019 (1): 7420781. doi: 10.1155/2019/7420781.

[27] LaValle, S.M. Virtual Reality. 2015. <http://lavalle.pl/vr/> [accessed Mar 31, 2021].

[28] -Valladares EH, Medellin-Castillo HI, Ballesteros J, Rodriguez-Florido MA. On the Development of Virtual Reality Scenarios for Computer-Assisted Biomedical Applications. J Healthc Eng. 2018;2018:1930357. 2018 Aug 30. doi:10.1155/2018/1930357

[29] Bundesverband Informationswirtschaft, Telekommunikation und neue Medien (Bitkom) e.V. Augmented und Virtual Reality: Potenziale und praktische Anwendung immersiver Technologien. 2021. <https://www.bitkom.org/Bitkom/Organisation/Gremien/Augmented-and-Virtual-Reality.html> [accessed Oct 21, 2021].

[30] Brennesholtz, M.S. 3-1: Invited Paper: VR Standards and Guidelines. 2018. <https://www.researchgate.net/publication/325488920_3-1_Invited_Paper_VR_Standards_and_Guidelines> [accessed Oct 21, 2021].

[31] Stach, M., Kraft, R., Probst, T., Messner, E.M.,Terhorst, Y., Baumeister, H., et al. Mobile Health App Database - A Repository for Quality Ratings of mHealth Apps. 2020. <http://dbis.eprints.uni-ulm.de/1913/> [accessed Oct 21, 2021].

[32] Yuan Y. Paving the Road for Virtual and Augmented Reality [Standards], IEEE Consumer Electronics Magazine, 7(1), 117-128. 2018. doi:10.1109/MCE.2017.2755338.
